# Subtyping Service Receipt in Personality Disorder Services in South London: Observational Validation Study Using Latent Profile Analysis

**DOI:** 10.2196/55348

**Published:** 2025-04-15

**Authors:** Jack Steadman, Rob Saunders, Mark Freestone, Robert Stewart

**Affiliations:** 1 Unit for Psychological Medicine, Wolfson Institute of Population Health Barts and The London School of Medicine and Dentistry Queen Mary, University of London London United Kingdom; 2 Centre for Outcomes Research and Effectiveness (CORE) Research Department of Clinical, Educational, and Health Psychology University College London London United Kingdom; 3 Institute of Psychiatry, Psychology and Neuroscience King's College London London United Kingdom

**Keywords:** latent profile analysis, latent variable mixture modeling, service use, personality disorder, applied health research, electronic health records

## Abstract

**Background:**

Personality disorders (PDs) are typically associated with higher mental health service use; however, individual patterns of engagement among patients with complex needs are poorly understood.

**Objective:**

The study aimed to identify subgroups of individuals based on patterns of service receipt in secondary mental health services and examine how routinely collected information is associated with these subgroups.

**Methods:**

A sample of 3941 patients diagnosed with a personality disorder and receiving care from secondary services in South London was identified using health care records covering an 11-year period from 2007 to 2018. Basic demographic information, service use, and treatment data were included in the analysis. Service use measures included the number of contacts with clinical teams and instances of did-not-attend.

**Results:**

Using a large sample of 3941 patients with a diagnosis of PD, latent profile analysis identified 2 subgroups characterized by low and high service receipt, denoted as profile 1 (n=2879, 73.05%) and profile 2 (n=1062, 26.95%), respectively. A 2-profile solution (*P*<.01) was preferred over a 3-profile solution, which was nonsignificant. In unconditional (*t*_3941,3939_=19.53; *P*<.001; B=7.27; 95% CI 6.54-8) and conditional (*t*_3941,3937_=−3.31; *P*<.001; B=−74.94; 95% CI −119.34 to −30.56) models, cluster membership was significantly related to receipt of nursing contacts, over and above other team contacts.

**Conclusions:**

These results suggest that routinely collected data may be used to classify likely engagement subtypes among patients with complex needs. The algorithm identified factors associated with service use and has the potential to inform clinical decision-making to improve treatment for individuals with complex needs.

## Introduction

Personality disorders (PDs) are typically associated with poorer health outcomes [1] and high mental health service use [2], yet PD treatments remain poorly understood and are often not specific to the individual [3]. Understanding patterns of service use across the diagnostic spectra of PD comprises an important and novel area of research in the applied domain. There are also considerations of optimizing UK National Health Service (NHS) service capacity and improving relationships between services and service users, which are often challenging given the relational sequelae component within a PD diagnosis [4].

The longstanding needs of patients with PDs require effective management and treatment approaches. Being able to understand likely patterns of service use could help allocate resources in secondary care, given limited resources. Predictive modeling for more personalized treatments is vanishingly rare in conventional health care models of service delivery [5], yet there are growing calls in the methodology and policy literature for improvements in both treatments and relationships [6] for patients with complex needs.

Effective prediction of treatment outcomes is an area of continued interest in general mental health care [7]. To our knowledge, this body of work has yet to be applied to PD treatments; however, there are promising opportunities offered by large-scale applied health data research, greater availability of relevant datasets [8], and increasing sophistication of relevant computer software and analytical approaches. This paper expounds formative work in this sphere by conducting formal validation in understanding diagnostic patterns of service receipt, as a key step toward formal prediction and more tailored treatments.

Given the engagement difficulties that come with a PD diagnosis [9], there is clinical interest in better understanding patterns of service use across the swathe of *International Classification of Diseases, Tenth Revision* (*ICD-10*) PD diagnoses [10,11]. Enabling services to predict likely patterns of service contact can help personalize the treatment pathway, given likely service demand, and optimize resource allocation. In turn, if treatment pathways can be tailored to the needs of service users at presentation, this may reduce the propensity for harmful treatments [12], directly improve therapeutic relationships, indirectly reduce long-term service use [13], and aid capacity management and resource allocation [14].

Individuals with a diagnosis of PD are reported as misunderstood, at least in mental health care [15]. In addressing this impasse, this study provides mental health services with the necessary functionality to move away from uniform PD treatments and toward more personalized approaches [16]. Decision support studies in health care are gaining momentum [17], and although the adoption of such systems has been slower in mental health care, the past decade has seen a rise in pilot implementations [18,19]. There is also a need for more advanced methodologies beyond “first-generation” regression-based approaches, which face increasing challenges and scrutiny in the age of data scientific [20], such as mediation and structural equation models, both in PD management [21] and in clinical psychology [22,23].

There has been growing interest in person-centered approaches [24] driven by calls for methodological and analytical advancements [25], efforts to improve access [26,27], initiatives to democratize treatments [28], and a rising focus on the personalization of mental health care [16]. These, in turn, have resulted in developments in theory [29] and modeling [30,31]. For example, latent class analysis (LCA) comprises an increasingly researched, novel approach, especially relevant in unpicking potential sources of observed heterogeneity [32,33]. This paper integrates these research perspectives using latent profile analysis (LPA). LPA is an extension of LCA, statistically described as finite Gaussian mixture models [34].

Person-centered approaches, such as clustering methods, LCA, and LPA, provide a useful first step in dimension reduction [35], argued as a key focus in reducing heterogeneity encountered in PD treatments, research, and theory [36]. In the context of psychiatric comorbidity, the number of potential co-occurring factors that make up disorders can challenge traditional variable-centered approaches, which may struggle to model all potential interactions [29]. In contrast, clustering approaches comprise an important first step in the aim to personalize treatments and treatment pathways, such as with physical medicine in cancer treatments [37]. In addition to these clinical and methodological motivations, the search for subgroups in complex mental health treatment is further motivated by relevant policy calls in the treatment literature [38].

The aim of this study was to characterize the patterns of service receipt in a large sample of patients with a diagnosis of PD in an urban, secondary care setting. The research question considered how to best characterize service receipt, as part of the specialist PD treatment pathway, using routinely available treatment, diagnostic, and demographic data.

## Methods

### Overview

LPA was conducted to identify statistically distinct patterns of service contact within a sample of individuals with a diagnosis of PD receiving treatment in secondary care in South London. In addition to identifying the optimal class solution in this setting, formal validation aimed to inform generalization estimation, reporting both average-level and individual-level classification errors. This paper further extends LPA research through consideration and evaluation of the structural model and extends such evaluation through protocolized resampling.

Validation-focused analyses assessed the structural model [39], with parameters tested for relevance when considering cluster membership. Out-of-sample approaches to prediction are well researched in wider structural equation modeling perspectives, for example, partial least squares structural equation modeling [40]. Importantly, while findings of mixture models may prove interesting, classes may not necessarily reside in the wider population [41], further motivating explicit consideration of generalization in study design.

**Figure 1:**
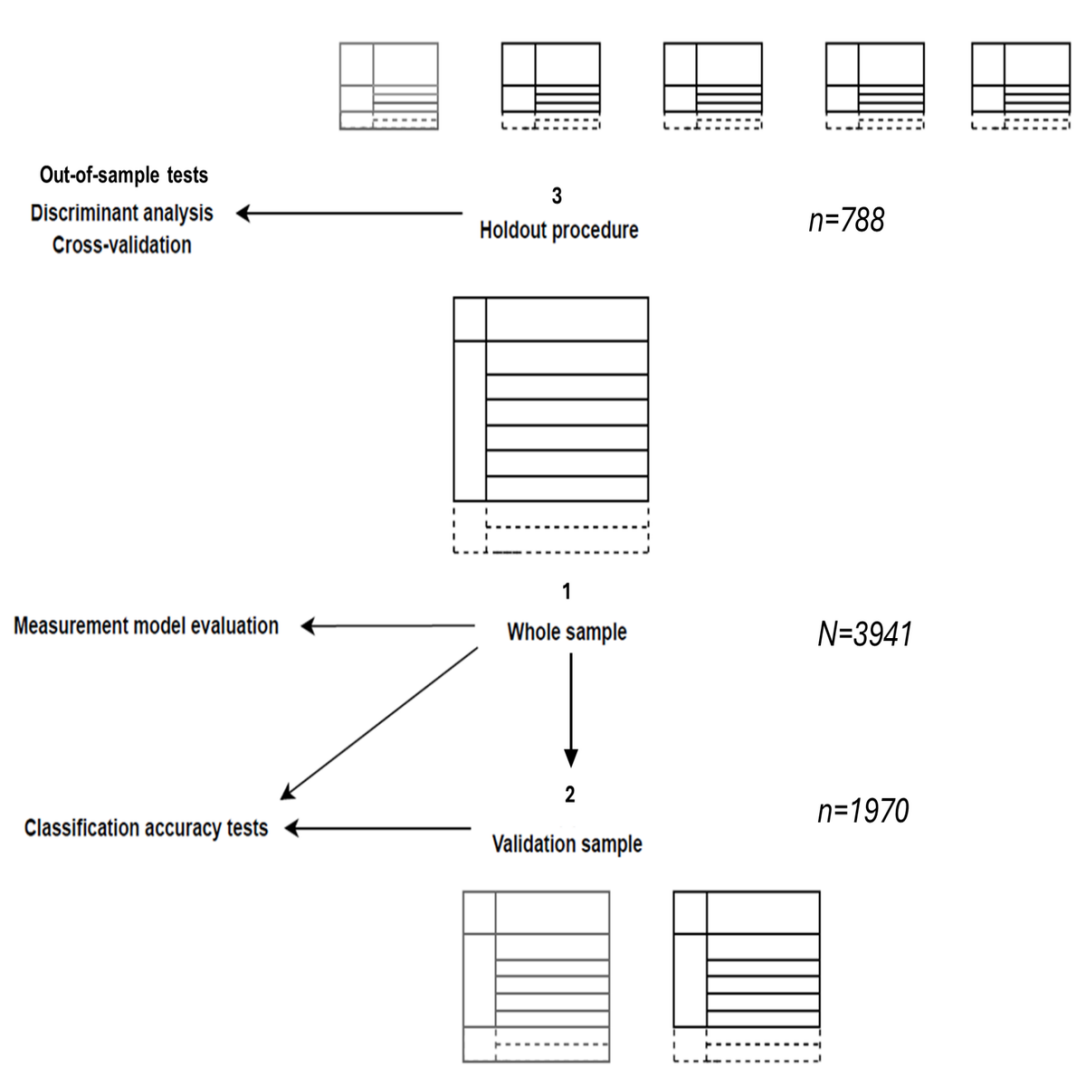
Analysis plan and sampling procedure.

LPA guidelines suggest using 3 sampling frames as good practice to validate findings [42,43]. To begin, the initial whole sample was “sample frame 1,” comprising the final analytical set. Validation samples randomly split the whole sample into two equal groups of 3941, resulting in “sample frame 2” [42,44] and 2 validation samples (1970/3941, 49.98%). Finally, cross-validation requires a holdout procedure [45], which partitions the dataset into test and training samples, to assess “out-of-sample” performance. This procedure split the whole sample into 5 parts, with 80% or *k−*4 parts designated as the training sample (3153/3941, 80%) and the remaining 20% or *k*−1 part designated as the test sample (788/3941, 20%). As shown in Figure 1, there were 3 sampling frames as a result.

In this study, posterior probabilities of cluster membership were identified using sample frames 1 and 2. The comparison between sampling frames (Tables S1 and S3 in Multimedia Appendix 1) describes the formal relevance of the model regarding validation. Posterior probabilities of cluster membership were calculated from the e-step of the expectation maximization algorithm [46] and describe how the person *i* belongs to the class *k* [47,48], also described as their modal class assignment, according to the selected model [42].

### Setting

Patient data for the study were extracted from the Clinical Record Interactive Search platform at the South London and Maudsley NHS Foundation Trust. South London and Maudsley (SLaM) provides comprehensive, near-monopoly specialist mental health services to a geographic catchment area of 4 boroughs of southeast London (Croydon, Lambeth, Lewisham, and Southwark) with a population of approximately 1.3 million residents.

### Ethical Considerations

The Clinical Records Interactive Search (CRIS) system was set up in 2007-2008 through codevelopment of a data security and governance model with service users [49]. The CRIS system and related linked data have received successive research ethics approvals since 2008 as an anonymized data resource for secondary analyses (current approval: Oxford Research Ethics Committee C, approval number 23/SC/0257). The institutionally approved governance model has been described in detail elsewhere [50], but in summary, it includes automated deidentification of the record, standard researcher approval, a locally publicized opt-out facility, and an oversight committee with patient leadership and membership reviewing all data use.

### Sample

The inclusion criteria comprised having an *ICD-10* PD diagnosis [41] at any time, being aged between 20 and 101 years at initial presentation, and being in contact with SLaM due to referral, although 10.38% (409/3941) of the sample had no recorded team contacts in the analysis set. Given the population of interest and generalization intention, those aged <20 years (318/3941, 8.07%) and >101 years (8/3941, 2.02%) were excluded to reduce possible inclusion bias and to encourage greater sample representativeness.

The inclusion criteria were applied over an exposure period of 11 years, with care episodes sampled between 2007 and 2018. This study sought to identify a matched, complete set of service use data at service users’ first and final points of contact with services (time 1 and time 2, respectively). Time points were denoted by episode start and end dates, such that time 1 represented the start and end of the first episodes of care and time 2 represented the same at the final episodes of care. Dataset assembly resulted in a complete, matched set of data points, ascribed by time 1 and time 2 and organized by episode of care. An episode of care was calculated as the elapsed time between patients being referred to and in contact with services and subsequent discharge from secondary services. In the final matched pairs set, the average length of an episode of care was 131.86 (SD 309.69) days. Moreover, “discharge” is defined by SLaM Trust policy as “discharging a patient from an in-patient setting to a community team or from the Trust completely to primary care or another provider” [51]. Importantly, this follows when a patient is deemed to no longer benefit from either reduced risk or improved well-being [51] and suggests a shift in presentation and overall needs.

Working with the CRIS team, a custom data extract resulted in 82,265 episodes of care from 8510 service users. The inclusion of contacts from clinical teams data resulted in a reduced set of 6068 individuals, which was further processed to include complete “matched pairs” data for 3941 service users, at their first and final episodes of care (T1 and T2, respectively; Figure S1 in Multimedia Appendix 3). A complete dataset was preferred as there were no missing data, which strengthened the design using a large sample. T1 and T2 contained episode start and end dates, respectively, as discussed in the Statistical Analysis section*.* The authors acknowledge that the nature of this experimental design necessitated service users engaging with services on more than one occasion. Therefore, individuals with only a single episode of care (2127/3941, 53.97%) were omitted from the analysis. Notwithstanding, PD is typically a chronic condition [52,53], and in general, service users are likely to have ongoing needs and therefore present to services on more than one occasion.

### Measurements

#### Quantitative Variables

Mixture modeling research typically uses cross-sectional data [54]. In the search for the best model, LPA compared model solutions using both repeated measures (T1 and T2) and T1 data only. Analytically, the episodic shaping of the data did not account for the timing or point of diagnosis, resulting in some patients being diagnosed after the initial T1 episode of care. Once again, however, given the longstanding nature of PD [52] and difficulties in accessing effective treatments [55], the precise timing of when a patient receives a diagnosis can be considered less important than it may initially appear.

Preliminary modeling [56] identified negligible “loading” or lambda values when including more granular detail regarding ethnicity (Table S1 in Multimedia Appendix 4). For example, including ethnicity categories such as Asian (λ=0.25) and mixed ethnicity (λ=−0.23) in the demographic construct failed to meet statistical significance, and the strongest performing model was the one that only included the presence or absence of White ethnicity.

Demographic data were modeled to include ethnicity (White or non-White), neighborhood-level deprivation, age, and gender (proportion of female and other). Deprivation was measured using the Multiple Indices of Deprivation [57]. This measure comprises nationally recorded data and measures multiple deprivation at the Lower Super Output Area level (a standard administrative geography encompassing approximately 1500 residents), including a nominal variable of 10 categories, with 1 describing most deprived and 10 describing least deprived [57].

Service receipt was modeled to include the total number of face-to-face contacts, as well as the number of unattended appointments, for each episode of care. Clinical input data were counted as the number of contacts from each treating team during each episode of care, that is, the cumulative total number of contacts within the specified time frame of a care episode. The number of contacts from care teams included medical, psychology, nursing, therapy, social work, and other. The CRIS system uses the General Architecture for Text Engineering [58], a natural language processing engine that captures recorded diagnostic statements. This output was used to ascertain PD diagnoses (Table S2 in Multimedia Appendix 4) in addition to prestructured primary diagnosis fields, which provide all options from the *ICD-10* [59].

*ICD-10* lists a range of PD diagnoses, denoted by category membership, in contrast to *ICD-11*, which characterizes PD by severity. Accumulating evidence indicates limited support for the foregoing polythetic approaches [11,60]. To aid meaningful comparisons, diagnoses were collated and assigned to relevant clusters used routinely in psychiatric care (Table S2 in Multimedia Appendix 4). Diagnostic and Statistical Manual cluster A diagnoses are characterized by “odd and eccentric” presentations, including paranoid, schizoid, and schizotypal PD diagnoses. These were distinguished from cluster B diagnoses, described as “overly dramatic, overly emotional, or unpredictable” presentations, as well as cluster C diagnoses, namely, “fearful or anxious” presentations.

As with ethnicity data, preliminary statistical modeling [56] highlighted the negligible impact on model performance of including more specific diagnostic information (Table S1 in Multimedia Appendix 2), beyond the presence or absence of cluster A diagnoses alone. Specifically, this was exemplified by low lambda loading values (λ<0.40) when including cluster B (λ=0.25), cluster C (λ=−0.20), and cluster other (λ=−0.07) onto the overall construct of diagnosis (refer to Tables S1 and S2 in Multimedia Appendix 4 for data cleaning descriptions of ethnicity and diagnostic data, respectively). Following these inferences, the proportion of *DSM* A diagnoses were taken as the proportion of *internalizing* diagnoses [61].

#### Statistical Methods

As formulated in the study by Nylund et al [62] and discussed in the study by Masyn [42], the null hypothesis in mixture modeling is the one-class model. Comparative likelihood ratio approaches, using the likelihood ratio test (LRT), describe and compare the likelihood of the two models under comparison, given the data [63]. In the first stage, different model solutions (namely, 2-, 3-, or 4-cluster solutions) were tested against this null sample. In the second stage, the number of groups, or “G,” was varied using *mclust* [35], and relevant likelihood ratio statistics were then entered into *tidyLPA* [64]. Second-stage testing specified the null (ie, the 2-class solution) versus the alternative (ie, the 3-class solution) hypothesis. The LRT statistic then compares the likelihood of either model, given the data [63].

Model evaluation then proceeds from the measurement model, in terms of number of classes, to the structural model, in terms of the relation of parameters to classes. Across the range of “stepwise” configurations, 1- and 3-step procedures are typical of the literature [39,62], with different approaches varying in their inclusivity and efficiency [65,66]. In practice, comparing the relationship between parameters and clusters can assist in model evaluation. These relationships describe the structural model of the mixture model, and structural model evaluation compares these relationships.

Discriminant analyses are popular in machine learning perspectives on mixture models [67,68]. Discriminant validity assesses the classification accuracy of a mixture model by comparing the performance of a testing subsample on a training subsample (Figure 1). In this study, discriminant analysis was developed using the eigenvalue decomposition discriminant analysis [35,41]. The eigenvalue decomposition discriminant analysis constrains the discriminant analysis model to have a single component for each class, with the same covariance structure among classes. More recent discriminant analysis models are available [69]; however, these models impose stricter requirements, such as a different number of mixture components and covariance structures for each class, which were deemed unsuitable for this analysis.

Discriminant analysis was conducted over 10 folds. For each “fold,” a separate dataset was assembled, comparing actual values versus those predicted by the model. As is typical in prediction studies, prediction error tests the predictive performance of the model on a holdout sample, with results summarized using common prediction metrics [45]. Typically, these include the root mean squared error, mean absolute error (MAE), and mean absolute percentage error (MAPE) [45,70]. In predictive applications, the MAE was selected as the metric of choice, given its greater resistance to distributional skewness [70]. To assist interpretation, both the MAE and MAPE are reported, with the MAPE calculated using the *Metrics* package [71]. The MAPE is calculated using the absolute error in each fold divided by the observed values that are evident for that fold. Averaging these fixed percentages yields the MAPE, which indicates how much error arises from the prediction model compared with the true value [72].

LPA was chosen for its flexibility in data requirements and its suitability for modeling a heterogeneous population that cannot be effectively represented by a single-component distribution [34]. Indicator variables included demographic variables, number of face-to-face contacts, number of did-not-attends, and number of contacts with clinical teams, per episode of care. Data preparation was assisted using the *tidyverse* package [73]. Analyses were conducted using the packages *mclust* [35] and *tidyLPA* [64] using R statistical software (R Core Team) [74,75].

### Statistical Analysis

#### Fitting the Measurement Model

The purpose of this analysis was to identify the patterns of service receipt in a large sample of patients with a diagnosis of PD in a secondary care setting, with the goal of enhancing contemporary models of care [76]. LPA scholarship was further extended through formal validation, as a precursor to prediction, which used protocolized resampling to ascertain the discriminatory relevance and possible scale potential of the proposed model solution.

To establish the best-fitting model, a range of metrics were considered. These included the Vuong-Lo-Mendall-Rubin LRT [77] and the bootstrapped LRT [39,78]. Alongside likelihood ratio tests, fit statistics included the Akaike information criterion, the Bayesian information criterion (BIC), and values of entropy. Both the LRTs, the Vuong-Lo-Mendall-Rubin LRT and bootstrapped LRT compare the *K* model (the current model with a number of *k* profiles) to *K*−1 model (a model with one less profile). Significant *P* values from these tests assert that the *K* model fits the data better than a comparable model with one less profile [43]. Conversely, a nonsignificant *P* value (*P*≤.05) indicates that the model with one less profile provides a better fit for the data, with more parsimonious models preferred. Smaller Akaike information criterion and BIC values indicate better model fit, while higher values of entropy suggest higher accuracy in classification of the model.

As no prior hypotheses for the number of profile groups were suggested, model selection compared solutions derived from either a repeated measures design, consisting of T1 and T2 data, or a contemporaneous design, consisting of T1 data only. Episodes of care were defined across two levels: (1) at the patient level, including demographic and diagnostic information, and (2) at the episode level, including “start” and “end” date of episodes, whereby service receipt measures were collected as properties, or descriptors, of that episode.

Episode descriptors were used as indicators for the LPA, including sociodemographic measures (age, ethnicity, and gender); diagnostic factors (percentage of internalizing diagnoses); general measures of contact (the number of face-to-face contacts and the number of did-not-attends); and the number of contacts from specific clinical teams (medical, nurse, psychology, therapy, and other).

#### Fitting the Structural Model

Assessment of the structural model considered the explanatory relevance of parameter values on cluster membership. Exogenous, pretreatment variables (ie, demographics) were limited to 1-step evaluation procedures. For service contact parameters, inferential tests were developed for both 1- and 3-step procedures [39]. Three-step procedures assert the importance of conditional probability of class membership in post hoc testing. Corrected by assignment probability [42], the 3-step procedure helps control for the individual validity of profiles. In contrast, the 1- and 2-step approaches can often omit the nonzero classification accuracy of derived clusters [39]. However, in the case of models with high classification accuracy, 1-step procedures may be justified [65,79]. As such, this paper reports both 1- and 3-step procedures, comparing unconditional versus conditional structural mixture models.

**Figure 2:**
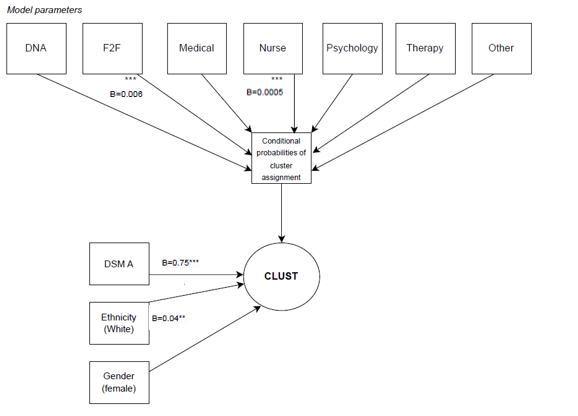
Conditional structural model evaluation showing the significance of paths and pretreatment covariates in determining cluster membership.

For the adapted 3-step procedure, the first step identified and enumerated cluster solutions, which were compared using model fit statistics. In the second step, conditional probabilities were extracted from the e-step of the EM algorithm using *mclust* [35] and vectorized “row wise” at the level of each individual. For the third step, individual conditional probabilities were included as covariates (ie, general linear models mapped the interaction between parameters with conditional probabilities). General linear models compared cluster assignment as a function of each parameter. Results of these analyses are summarized in Figure 2 and presented in Table 1.

**Table 1:** Direct and controlled effects of cluster membership and additional covariates on parameters.

		*t* test (*df*)	Direct effect, 95% CI	*t* test (*df*)	Controlled effect (95% CI)
**Engagement**					
	Did-not-attend	19.05 (3941,3939)	4.30 (3.85 to 4.74)	0.05 (3941,3937)	2 (4 to 10)
	Face to face	28.70 (3941,3939)	31.33 (29.19 to 33.47)	−1.04 (3941,3937)	−68.86 (−199.13 to 61.41)
**Clinical contacts**					
	Medical	23 (3941,3939)	4.91 (4.49 to 5.33)	−1.30 (3941,3937)	−16.89 (−42.38 to 8.60)
	Nurse	19.53 (3941,3939)	7.27 (6.54 to 8)	−3.31^a^ (3941,3937)	−74.94 (−119.34 to −30.56)
	Psychology	23.83 (3941,3939)	5.71 (5.24 to 6.18)	1.72 (3941,3937)	25.01 (−3.57 to 53.59)
	Therapy	15.37 (3941,3939)	3.21 (2.80 to 3.61)	1.07 (3941,3937)	13.57 (−11.35 to 38.49)
	Other	12.64 (3941,3939)	4.84 (4.09 to 5.59)	0.45 (3941,3937)	10.60 (−35.14 to 56.34)

^a^*P*<.001.

### Latent Profile Cross-Validation Strategy

Cross-validation uses the data to test “the predictive power” of the model [80]. For the procedure, cross-validation splits the dataset into k subsamples of equal size, repeating k times, each time using a different test set (Figure 1). The best-fitting parameters of the model are then fit using k−1 subsamples, with the model’s error then computed on the unused subsample or test set [81].

In line with the discriminant analysis procedure, cross-validation was conducted using sample frames 1 and 2 (in sample and out of sample, respectively), as shown in Figure 1. The Brier score [82,83] indicates the predictive usefulness of the model for individuals who, theoretically, may be unseen. Applied research in mental health has shown increased experimentation with the Brier score as a forecasting tool, such as for predicting suicidal ideation in individuals at near-term suicide risk [84] or identifying patients likely to drop out of psychotherapy [85]. In related psychotherapy research, the Brier score ranges from 0 (best prediction) to 1 (worst prediction), and it measures the accuracy of probabilistic predictions [85].

A detailed sampling strategy enabled both explanatory and predictive appraisal of the derived model solution, as shown in Figure 1. As well as the whole sample (frame 1), validation samples (frame 2) divided the dataset randomly, 2 × 50%. LPA was conducted on both frames 1 and 2 (total sample and validation samples, respectively), as shown in Figure 1, informing sample comparisons and confirming the profile structure [43]. Subsequently, LPA was rerun on the whole sample to aid interpretation of the findings.

Sample frame 3 for discriminant analyses split the total sample into 5 parts, with 80% (3153/3941) or *k*−4 parts designated as the training sample and the remaining 20% (788/3941) or *k*−1 part designated as the test sample. The holdout procedure was repeated over 10 folds, in line with best practices [45]. Discriminant analyses tests were then applied over sampling frames 1 and 3 (Figure 1), with cross-validation describing classification accuracy for both total and holdout sampling frames using the classification error and Brier scores. As discussed earlier, the Brier score is a proper score function that measures the accuracy of probabilistic predictions [85]. More technically, the Brier score comprises the mean square difference between the true classes and predicted probabilities [82,83].

### Latent Profile Resampling Testing Strategy

Discussions on prediction-focused structural equation modeling consider discriminant validity in terms of in-sample versus out-of-sample perspectives [45]. Bootstrapping has gained prominence in sample validation; however, this remains a decidedly in-sample approach. In this study, larger sample sizes permitted a combination of k-fold assessments of discriminant validity, combined with cross-validation modeling, which ascertain both in-sample and out-of-sample classification accuracy.

LPA was then conducted on the “T1 and T2” dataset, while a holdout procedure (Figure 1) tested the predictive relevance of the profile solution across 10 partitions, or “folds,” of this procedure. Predictive performance was summarized by prediction metrics, namely, the MAE and MAPE [86,87]. The MAPE was calculated using the absolute error in each fold divided by the observed values that are evident for that fold. Averaging these fixed percentages yields the MAPE, which indicates how much error arises from the prediction model compared with the true value [72].

## Results

This study broadly follows the reporting guidelines from the Consolidated Standards of Reporting Trials (CONSORT) [88], although it specifically adheres to the Strengthening the Reporting of Observational Studies in Epidemiology [89] and Reporting of Studies Conducted Using Observational Routinely-Collected Data [90] guidelines, which were more suited for improving the reporting of observational epidemiological research.

### Participants

The flow of participants throughout the study is shown in Figure S1 in Multimedia Appendix 3. In brief, the initial analytical set comprised 82,265 episodes and 8510 patients. Data were prepared to include team contacts and ensure completeness, resulting in 170,365 episodes and 6421 patients. Further processing resulted in the final analytical set of 7882 episodes from 3941 patients to optimize the analysis, with this complete dataset having no missing values.

### Descriptive Statistics

Tables 2 and 3 display the distribution of patient characteristics from the LPA and across sampling frames. Descriptive statistics for holdout samples were limited to the initial fold (of 10). Comparing latent profiles to the whole sample means and distribution provides an understanding of the characteristics of each group of patients across identified latent profiles (LPs)—LP1 and LP2—in the 2-profile solution. Service receipt, measured as the number of contacts from clinical teams, was markedly higher for LP2 than for LP1. The LPA best characterized service receipt as being either high or low, captured through LP2 and LP1, respectively. While LP1 counted no psychology contacts, LP2 on average had almost 6 per episode of care. This pattern was consistent for medical teams and, in particular, nursing teams.

In general, LP2 received treatment at a rate 10 times higher than LP1. The derived profiles were not differentiated by sociodemographic characteristics; both latent profiles had similar proportions of female and White individuals as well as comparable levels of neighborhood deprivation. In total, 11.3% (120/1062) of LP2 had internalizing diagnoses, with no such diagnoses present in LP1.

**Table 2:** Latent profiles and associated patient characteristics for the full sample; indicator values for sample frames 2 and 3.

			Sample frame 1 (N=3941)		LP1^a^ (n=2879; 73.05%)	LP2 (n=1062; 26.95%)		Sample frame 2		Sample frame 3 (holdout)	
								Validation sample a (n=1970)	Validation sample b (n=1970)	Train (n=3153)	Test (n=788)
**Demographics**											
	Age (y), mean (SD)	44.33 (14.30)		44.10 (14.1)		44.95 (14.90)	43.90 (14.23)		44.38 (14.29)	44.49 (14.26)	43.68 (14.41)
**Gender** **(n=2482, 63%)**											
	Female, mean (SD)	2482.8 (1891.7)		1805.1 (1381.9)		660.6 (1062)	1221.4 (965.3)		1241.1 (945.6)	1986.4 (1513.4)	472.8 (386.1)
**Deprivation**											
	IMD^b^, mean (SD)	3.68 (1.73)		3.66 (1.75)		3.70 (1.67)	3.74 (1.76)		3.64 (1.65)	3.66 (1.69)	3.74 (1.87)
**Ethnicity (n=1261, 32%)**											
	White, mean (SD)	1261.1 (1852.3)		878.1 (1324.3)		386.6 (509.8)	650.1 (925.9)		650.1 (925.9)	1008.9 (1481.9)	252.16 (370.4)
**Diagnostic data**											
	Internalizing (y), mean (SD)	118.23 (669.9)		0 (0)		120 (339.8)	59.1 (315.2)		59.1 (354.6)	94.59 (536)	23.64 (126.08)

^a^LP: latent profile.

^b^IMD: indices of multiple deprivation.

**Table 3:** Latent profiles (LPs) and associated engagement characteristics across sampling frames; indicator values for sample frames 2 and 3.

		Sample frame 1 (N=3941)	LP1 (n=2879; 73.05%)	LP2 (n=1062; 26.95%)	Sample frame 2		Sample frame 3 (holdout)	
					Validation sample a (n=1970)	Validation sample b (n=1970)	Train (n=3153)	Test (n=788)
**Engagement, mean (SD)**								
	F2F^a^	12.88 (33.44)	4.43 (5.17)	35.78 (58)	12.06 (30.25)	13.03 (33.93)	12.93 (33.49)	12.65 (33.24)
	Did-not-attend	1.59 (6.57)	0.43 (1.03)	4.73 (12)	1.57 (6.51)	1.59 (6.79)	1.60 (6.62)	1.55 (6.35)
**Clinical team input, mean (SD)**								
	Medical	2.32 (6.33)	1 (1.50)	5.90 (11.2)	2.20 (6.13)	2.33 (6.78)	2.32 (6.17)	2.34 (6.94)
	Nurse	4.11 (10.86)	2.15 (3.36)	9.43 (19.20)	3.98 (10.23)	4.14 (10.13)	4.13 (11.41)	4.05 (8.29)
	Psychology	1.54 (7.13)	0 (0)	5.70 (12.90)	1.49 (7.01)	1.46 (6.76)	1.64 (7.57)	1.15 (4.98)
	Therapy	0.91 (5.98)	.05 (0.21)	3.25 (11.20)	0.74 (3.79)	0.94 (6.52)	0.89 (5.36)	0.99 (8.01)
	Other	1.56 (10.88)	0.25 (0.72)	5.10 (20.5)	1.23 (9.27)	3.64 (1.65)	1.55 (11.09)	1.60 (9.99)

^a^F2F: face to face.

### Evaluation of the Measurement Model

Analyses identified 2 profiles emerging from the contemporaneous (T1) dataset and 4 profiles from the repeated measures dataset (T1 and T2; Table 4). Profiles were enumerated according to the BIC using mclust [35] for T1 versus T1/T2 dataset, selecting 2 and 4 profiles, respectively. The comparison of classification and likelihood indices shown in Table 4 suggested that the 2-profile solution from T1 data was preferred, showing superior model fit. Stage-1 LRT tests compared 2 and 3 class solutions against the null hypothesis, that is, the 1-class model. Both solutions demonstrated superior likelihood than the null hypothesis; however, the 2-profile solution was preferred. Stage-2 LRT tests compared 3-profile (the alternative) versus 2-profile (the null) solutions and failed to demonstrate any advantage of a 3-profile solution over a 2-profile solution.

**Table 4:** Model fitting statistics^a^.

Fit values		1-cluster	2-cluster	3-cluster	4-cluster
**Log-likelihood criteria**					
	Log likelihood	−119,761	−72,497.47	−81,918.54	−118,721.9
	BIC^b^	−240,267.2	−146,402.4	−164,267.6	−245,317.3
**Classification criteria**					
	Entropy	—^c^	13.14	90	29
	Normalized entropy difference	—	0.034	0.025	0.0052
**LRTs** ^d^		1 versus 2	2 versus 4	3 versus 1	3 versus 2
	VLMR-LRT^e^	90,868.55^f^	−90,624.52	92,661.71^f^	ns^g^
	B-LRT^h^	94,527.1^i^	—	—	—

^a^Multivariate indicators: multivariate skewness=1425.64 (statistic 936,405.6); multivariate kurtosis=2840.37 (statistic 4576.15). For a formal mathematical definition, see Figure S2 in Multimedia Appendix 3.

^b^BIC: Bayesian information criterion.

^c^Not applicable.

^d^LRT: likelihood ratio test.

^e^VLMR-LRT: Ven-Lo-Mendall-Rubin likelihood ratio test.

^f^*P*<.001.

^g^ns: nonsignificant.

^h^B-LRT: bootstrapped likelihood ratio test.

^i^*P*<.01.

In model selection, comparative LRTs described how a 3-cluster solution significantly outperformed the 1-class model but not the 2-class model (Table 4); therefore, a 2-cluster solution was preferred.

Posterior probabilities were calculated as the proportion of correctly assigned patients to the respective clusters. These probabilities are “posterior,” in the sense that they are calculated *after* individuals have been assigned a cluster membership [91]. Table S2 in Multimedia Appendix 1 displays classification indicators and posterior probabilities, and the classification error of the model was also negligible, being near 0. The Brier scores further demonstrate near-perfect classification accuracy of the model in forecasting individuals’ likely engagement subtype, given the values of routinely collected indicators. High posterior probabilities are also considered clear indicators of classification accuracy, with values ideally exceeding 0.80, in line with established guidelines [66].

### Evaluation of the Structural Model

Findings from evaluation of the measurement model, taken with the near-perfect degree of classification accuracy (Table S3 in Multimedia Appendix 1), suggest the usefulness of combined parameters in predicting cluster membership.

Relating parameter values to clusters is expressed in the language of direct and controlled direct effects [92]. The direct effect describes the (unconditional) relationship between parameters and clusters, while the controlled direct effect describes this relationship based on the individual probabilities of cluster assignment. In the unconditional model, all parameters determined class assignment, apart from gender (Table 5). Figure 2 displays conditional model evaluation, where face-to-face contacts (*t*_3940,3939_=5.73; *P*<.001; B=0.006; 95% CI 0.005-0.006) and the number of nursing contacts (*t*_3940,3939_=5.52; *P*<.001; B=0.0005; 95% CI −0.003 to 0.0006) significantly determined class assignment.

**Figure 3:**
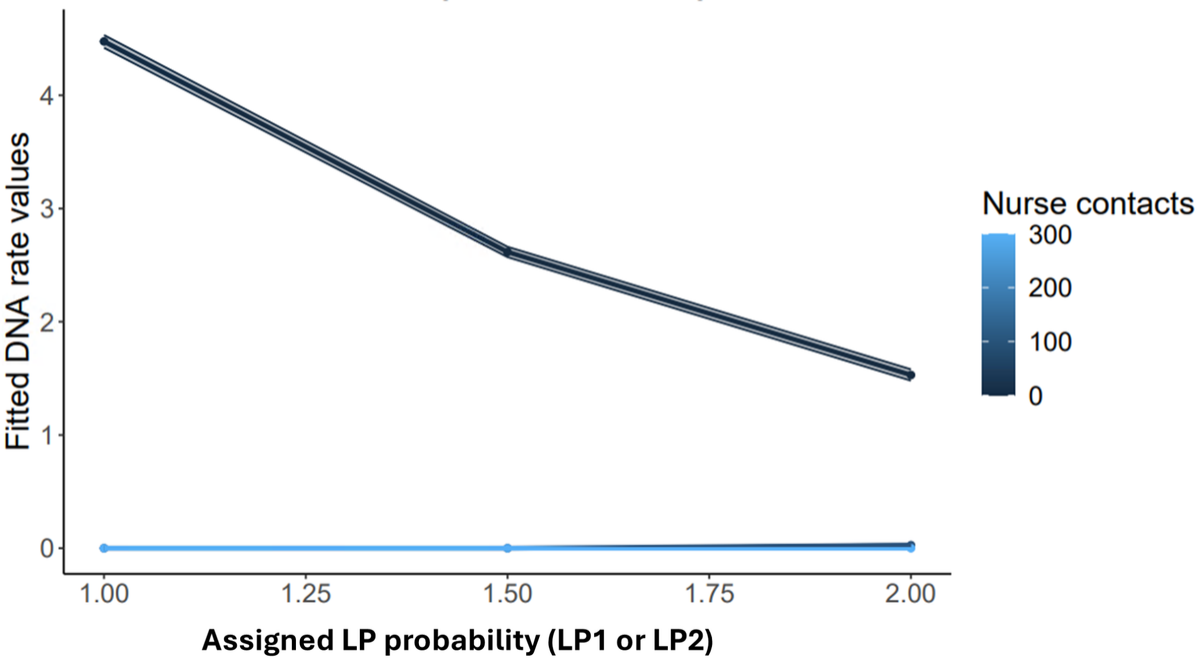
Plot showing the interaction term between nursing contacts and cluster in determining the rate of did-not-attends (DNAs).

Likelihood-based approaches tend to dominate the mixture modeling landscape; however, there can be inferential challenges when adhering to the orthodoxy [63,93]. A more Bayesian approach may consider the likely parameter value, given the cluster membership. Structural model evaluation using this approach followed the same 3-step procedure as advised by Nylund-Gibson et al [39] and described in the section Fitting the Structural Model. Results from these analyses are presented in Table 1 and further detailed in Figure 3.

In the unconditional model and of all care contacts, psychology demonstrated the strongest relationship to clusters (*t*_3940,3937_=23.83; *P*<.001; B=0.02; 95% CI 0.02-0.02); however, this was not upheld in the conditional model (*t*_3940,3937_=1.72; *P*=.09). Overall, key prognostic targets of service receipt given the knowledge of cluster membership were nurse contacts. In both unconditional (*t*_3940_=19.53; *P*<.001; B=7.27; 95% CI 6.54-8) and conditional (*t*_3939_=5.52; *P*<.001; B=−74.94; 95% CI −119.34 to −30.56) models, cluster membership was significantly related to the receipt of nursing contacts.

Further testing modeled an interaction term between nursing contacts and cluster membership in determining nonattendance, as shown in Figure 3. The interaction term was significant both for face-to-face contacts (*t*_3940,3937_=5.81; *P*<.001; B=0.74; 95% CI 0.49-0.99) and did-not-attends (*t*_3940,3937_=3.62; *P*<.001; B=0.12; 95% CI 0.06-0.20), with confirmatory testing indicating that the rate of did-not-attends did not significantly differ from a Poisson distribution (N=3941, χ^2^_8_=10.3; *P*=.20).

**Table 5:** Direct and controlled effects of parameters and additional covariates on cluster membership.

			*t* test (*df*)	Direct effect, 95% CI	*t* test (*df*=3939)	Controlled effect, 95% CI
**Engagement**						
	Did-not-attend		2.93 (3940)	0.004 (0.001 to 0.006)	1.53	0.0004 (−0.0001 to 9.86)
	Face to face		20.76 (3940)	0.005 (0.004 to 0.006)	5.73^a^	0.006 (0.005 to 0.006)
**Clinical contacts**						
	Medical		23 (3940)	0.024 (0.022 to 0.026)	1.38	0.0003 (−0.0001 to 0.0007)
	Nurse		19.53 (3940)	0.01 (0.01 to 0.01)	5.52^a^	0.0005 (−0.003 to 0.0006)
	Psychology		23.83 (3940)	0.02 (0.02 to 0.02)	1.72	0.0003 (−0.001 to 0.003)
	Therapy		15.37 (3940)	0.02 (0.02 to 0.02)	0.15	0.0002 (−0.002 to 0.003)
	**Other: sociodemographics**					
		Internalizing diagnoses	19.07 (3940)	0.75 (0.68 to 0.81)	—^b^	—
		Ethnicity (White)	2.97 (3940)	0.04 (0.01 to 0.06)	—	—
		Gender (female)	1.16 (3940)	0.001 (−0.01 to 0.04)	—	—

^a^*P*<.001.

^b^Not applicable.

Figure 3 shows this relationship visually and indicates how did-not-attend rates vary between clusters as a function of nursing contacts. The assignment of profiles is shown probabilistically between either latent profile 1 and latent profile 2, which allows for assignment likelihood or uncertainty in clinical practice. For LP2, the number of nursing contacts made less of a difference in fitted rates of did-not-attends, compared to LP1. As it represented the majority of the sample, LP1 was more sensitive to the effect of nursing contacts regarding fitted rates of attended appointments.

### Resampling Performance

Discriminant analysis modeling indicated high classification accuracy of the selected model (Table S3 in Multimedia Appendix 1). Both the MAE and MAPE were taken as summative indicators regarding the overall prediction error of the model. Over 10 folds of the holdout procedure, or train × test runs, the “grand” MAE was 0.004 (Table S3 in Multimedia Appendix 1), meaning that the model predicted correct class assignment with near-perfect accuracy. Conversely, out-of-sample tests on the replicative utility of engagement measures were less favorable. For face-to-face contacts, the MAE was 11.27, whereas for the number of did-not-attends, the MAE was 1.97. The MAPE may aid interpretation of these results, specifying that the number of face-to-face contacts erroneously predicts cluster membership in 11% (86/788) of cases (of 10 “folds”; Table S2 in Multimedia Appendix 1).

In the explanation-focused in-sample approach, the Brier score was 0.005. From an out-of-sample perspective, the average Brier score was 0.19 (Table S1 in Multimedia Appendix 1). The average classification error over 10 folds was 0.005, indicating substantially high classification accuracy. In addition, the average (out-of-sample) Brier score was 0.19 (SE 0.005), suggesting a nearly 80% rate of forecasted classification accuracy from an out-of-sample perspective.

## Discussion

### Summary of Findings

This study aimed to characterize the patterns of service receipt in a large sample of patients with a diagnosis of PD in an urban, secondary care setting. The main findings of this study were identification of 2 LPs that best characterize service receipt in specialist PD treatments. LP2, at 26% (1062/3941) of the sample, received almost 5-fold as many nursing contacts as LP1 (9.43 vs 3.98) and 6-fold as many medical contacts (5.90 vs 1). In addition, general service use was almost 10 times greater in LP2 than LP1. Sociodemographic characteristics were not associated with LPs, but diagnostic factors were associated. Specifically, LP1 had no internalizing diagnoses. These findings suggest important distinctions of service receipt as a function of routinely collected data and suggest the discriminatory relevance of such descriptors in determining likely profile membership.

LPA identified statistically reliable categories of service use using routine observational data for those with a diagnosis of PD. Profiles distinguish service receipt for those with a diagnosis of PD and were replicated across a range of sampling frames. Discriminant analyses and cross-validation modeling suggest substantial discriminatory performance in secondary settings using these types of data. This study described 2 LPs, primarily distinguished by diagnostic information. Of the approximately 3000 patients in LP1 (n=2879, 73.05%), none had internalizing PD diagnoses. In inferential testing, patients in LP1 were twice as likely to have less service contact.

### Interpretation

The literature discussing PD and service use describes greater service use in the general presence of PD [94,95]; however, this has yet to be robustly applied between different PD diagnoses. One study delineates the interplay between personality (disorder) and health [96], and there is rigorous work, such as comparisons of service use based on ethnicity in PD treatments [97]. Despite economies of scale offered by population-based interventions, these programs should be customized for subgroups on the basis of personality [96]. This study details foundational work in this area using a large sample in secondary care and attempts to export this comparative focus to diagnostic factors within PD treatments.

In a large study using administrative data in Quebec, emergency department visits were compared between “externalizing” or *Diagnostic and Statistical Manual* cluster B PD and schizophrenia, those diagnosed with both conditions, and the general population [98]. General practitioner visits were highest in the cluster B group, while the use of psychiatric (vs nonpsychiatric specialist or generalist) services differed in the presence of a PD diagnosis, with 1 mean annual visit versus 4, for schizophrenia.

Similar findings were captured in this study, which demonstrates lower service use for noninternalizing diagnostic groups. Greater service use was shown for the smaller latent profile, LP2. In practice, greater patterns of service use from the minority group arguably have predictive relevance in profile determination and tailoring treatment pathways accordingly. “Hotspotting,” as it is known in general practice [99], is a data-driven practice that aims to identify patterns of “extreme” use [100]. Complex needs and PD populations are similarly characterized by high use [101]. This responsive concept is well known in general practice but has yet to be specifically applied to mental health care. In the case of PD and complex needs, general practice is often a key contact [102]. As demonstrated in this study, understanding individual (stratified) patterns of service use offers an example for expanding hotspotting practices, commonly used by general practitioners, to address historical challenges in generalist service provision for complex needs. By applying advanced techniques such as LPA, this approach can help create more tailored treatment pathways.

Therefore, this study argues that more data-enabled, proactive service provision, engendered by hotspotting practices, may assist modernization of mental health provision and encourage more individually intelligible and tailored treatment pathways. The assertion is that the determination of cluster membership, given the routinely measured parameters, may help inform similar practices in secondary care with complex needs. Related work in improving access to psychological therapies highlights 8 LPs [43], which contrasts with the solution presented in this study.

Interestingly, the rates of nonattendance were stable across profiles, averaging around 1 in 10, which is broadly in line with the literature in general practice [103]. Service use was poorly distinguished as a function of the treating team. Structural evaluation revealed nursing contacts and did-not-attends of key interest; modeling the interaction term showed that the fitted rate of did-not-attends differs between clusters as a function of nursing contacts. The implication is that more intensive treatment pathways with more nursing contacts result in greater fitted nonattendance for LP1. However, for LP2, greater nursing contacts had much less of an effect on did-not-attends.

The use of a large sample, the multiplicity of analyses, and the development of a protocolized resampling scheme for validation comprise key strengths of this study. Discriminant validity and classification accuracy tests showed near-perfect classification for individuals with a diagnosis of PD into LPs (Table S2 in Multimedia Appendix 1). Generalizability was explicitly considered pursuant to resampling and validation, which may have implications regarding wider (national) policy implementation. From an out-of-sample perspective, for example, the model accurately predicted cluster assignment in nearly 80% of cases, across 10 “folds” of the holdout procedure, or train × test runs, compared with a Brier score of 0.005 for in-sample model performance (Table S1 in Multimedia Appendix 1). At least, for explanatory purposes, the model was able to classify all cases correctly.

### Limitations

A limitation of this study is that both exposure and outcome were measured over the same periods. This contemporaneous design was indicated in model selection; however, more frequent measurement may have resulted in more precise model solutions. Comparative modeling between both T1 and T1/T2 data suggested that a 2-cluster solution using T1 measurement showed superior model performance. In addition, the analytical design only included those who had more than 1 episode of care, which may exclude more sporadic or crisis presentations. Covariates were also drawn over time, rather than at presentation, rendering this study more suitable for validation than for strict prediction.

In addition, clinical outcomes were not assessed in this study, although there is scope to include these in future research. Finally, the direction of causality remains unclear—specifically, whether service receipt determines latent profile, or vice versa—along with the relationship between service receipt, profile, and clinical outcomes.

### Conclusions

Truly “predictive” models of care are currently uncommon in mental health [5]. However, this study plays an important role in moving toward more predictive models of care in the medium term, following the formal validation of LPs and deployment of a rigorous resampling procedure in testing classification uncertainty at both average and individual levels. Through the identification and description of LPs, services are envisioned to be equipped to include such information in more personalized treatment approaches. More reflexively, such approaches can be seen as facilitating preventative models of care rather than reactive ones [104,105], representing a radical and, if not timely, necessary shift in care delivery.

## Appendix

Multimedia Appendix 1 Additional validation and classification indices.

Multimedia Appendix 2 Ethnicity and diagnostic data of total sample.

Multimedia Appendix 3 Additional visual displays.

Multimedia Appendix 4 Data cleaning procedures for categorical data.

## Abbreviations

BIC: Bayesian information criterion
CONSORT: Consolidated Standards of Reporting Trials
CRIS: Clinical Records Interactive Search
ICD-10: International Classification of Diseases, Tenth Revision
ICD-11: International Classification of Diseases, Eleventh Revision
LCA: latent class analysis
LP: latent profile
LPA: latent profile analysis
LRT: likelihood ratio test
MAE: mean absolute error
MAPE: mean absolute percentage error
NHS: National Health Service
NIHR: National Institute for Health Research
PD: personality disorder
SLaM: South London and Maudsley

## Data Availability

The datasets generated or analyzed during this study are not publicly available.
