# Supplementary material for: Subtyping Service Receipt in Personality Disorder Services in South London: Observational Validation Study Using Latent Profile Analysis
Source: Interact J Med Res. 2025 Apr 15;14:e55348. doi: 10.2196/55348 (PMC12041827; doi:10.2196/55348)
Supplement: Multimedia Appendix 1 [file ijmr_v14i1e55348_app1.docx]

### Multimedia Appendix 1: Additional validation and classification indices

Table S1: Cross validation metrics. Out-of-sample displays mean values over 10 reps.

| **Cross validation statistics** | In-sample | Out-of-sample |
| --- | --- | --- |
| Classification error (SE*^a^*) | .005 | .20 (.006) |
| Brier score (SE) | 0 | .19 (.005) |

*^a^*SE = Standard Error

Table S2: Classification table of two-cluster solution using validation and full sample

| Sample frame | |  | *n^a^* | PP*^b^* | CE*^c^* | Brier score |
| --- | --- | --- | --- | --- | --- | --- |
| **Validation sample (n = 1,970)** | | |  |  | **0** | **0** |
| **Cluster (derived / assigned)** | | |  | |  |  |
|  | 1 | 1 | 1435 | 1 |  |  |
|  | 2 | 2 | 535 | 1 |  |  |
|  | 1 | **2** |  | 0 |  |  |
|  | 2 | **1** |  | 0 |  |  |
|  |  |  |  |  |  |  |
| **Whole sample (N = 3,941)** | | |  |  | .005 | .005 |
| **Cluster (derived / assigned)** | | |  |  |  |  |
|  | 1 | 1 | 2879 | .994 |  |  |
|  | 2 | 2 | 1062 | .995 |  |  |
|  | 1 | 2 |  | .005 |  |  |
|  | 2 | 1 |  | .005 |  |  |

*^a^n =* Sample size

*^b^*PP =Average posterior probability

*^c^CE =* Classification error

Table S3: Out-of-sample indicators for validation of latent profiles and parameters

| Parameters | *MAE^a^* | *MAPE^b^* |
| --- | --- | --- |
| Cluster | .004 | 0 |
| F2F*^c^* | 11.27 | 9.96 |
| DNA*^d^* | 1.97 | 1.35 |

*^a^*MAE = Mean Absolute Error,

*^b^*MAPE = Mean Absolute Percentage Error,

*^c^*F2F = Number of face-to-face contacts;

*^d^*DNA = Number of Did-Not-Attends
