# Supplementary material for: Subtyping Service Receipt in Personality Disorder Services in South London: Observational Validation Study Using Latent Profile Analysis
Source: Interact J Med Res. 2025 Apr 15;14:e55348. doi: 10.2196/55348 (PMC12041827; doi:10.2196/55348)
Supplement: Multimedia Appendix 2 [file ijmr_v14i1e55348_app2.docx]

Table S1: Ethnicity and diagnostic data of total sample 7,882 (T1 and T2)

|  |  | n | Proportion (%) |
| --- | --- | --- | --- |
| **Ethnicity** | |  |  |
|  | Mixed | 1046 | 13.3 |
|  | White | 5354 | 67.9 |
|  | Black | 1181 | 14.9 |
|  | Asian | 301 | 3.8 |
| DSM-IV Cluster | |  |  |
|  | Cluster A | 240 | 3.04 |
|  | Cluster B | 5112 | 64.9 |
|  | Cluster C | 323 | 4.1 |
|  | Other | 2207 | 28 |
