# Supplementary material for: Subtyping Service Receipt in Personality Disorder Services in South London: Observational Validation Study Using Latent Profile Analysis
Source: Interact J Med Res. 2025 Apr 15;14:e55348. doi: 10.2196/55348 (PMC12041827; doi:10.2196/55348)

### Multimedia Appendix 3: Additional visual displays

Figure S1: Participant flow throughout data preparation


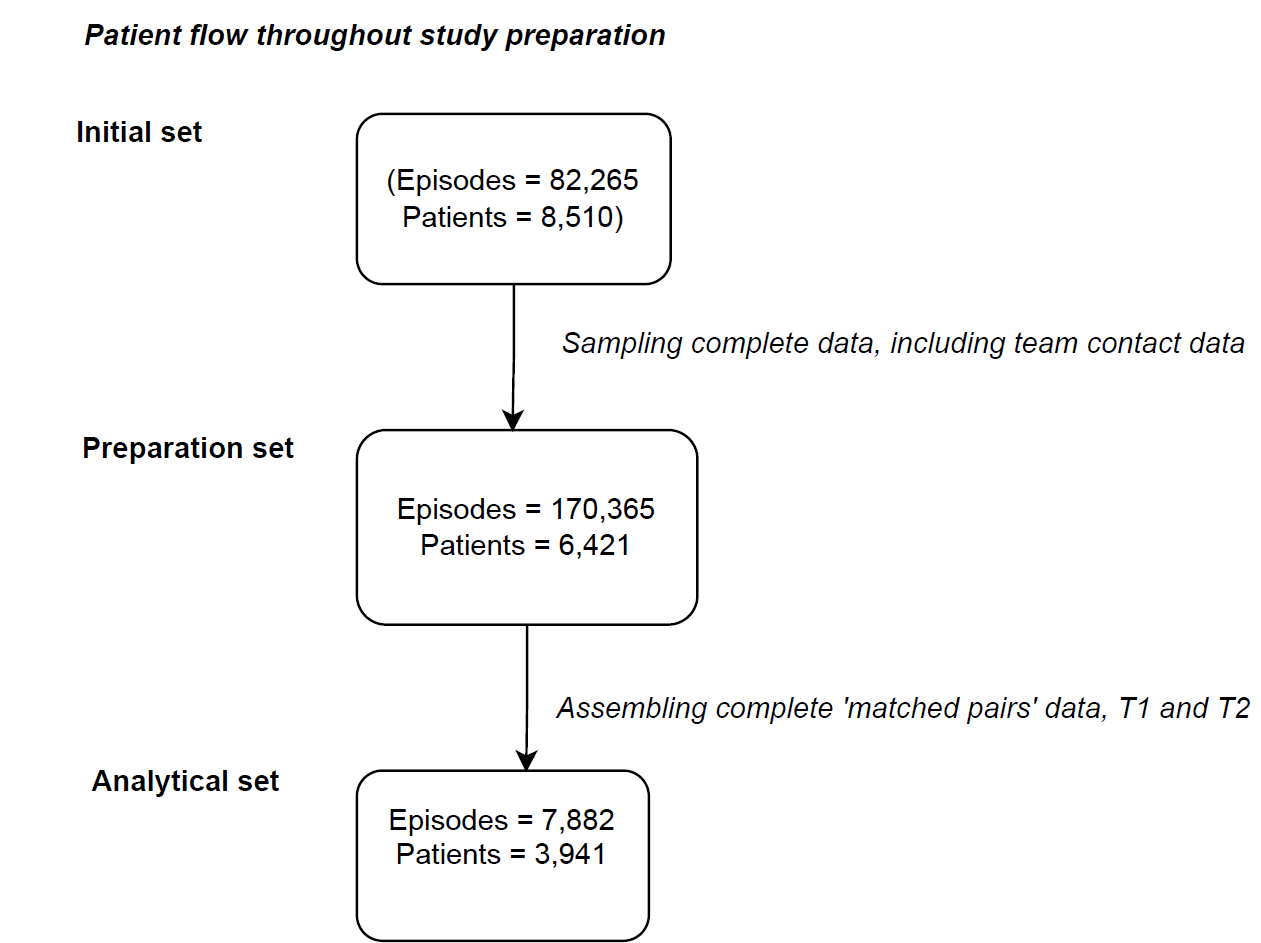


Figure S2: Formal mathematical definition of B1P (Multivariate skewness) and B2P (Multivariate kurtosis)


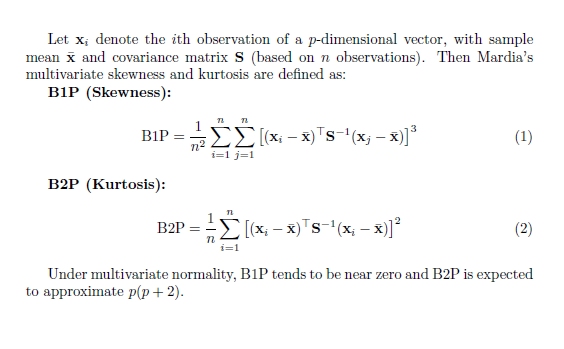


Figure S3: Service utilisation by latent profiles.


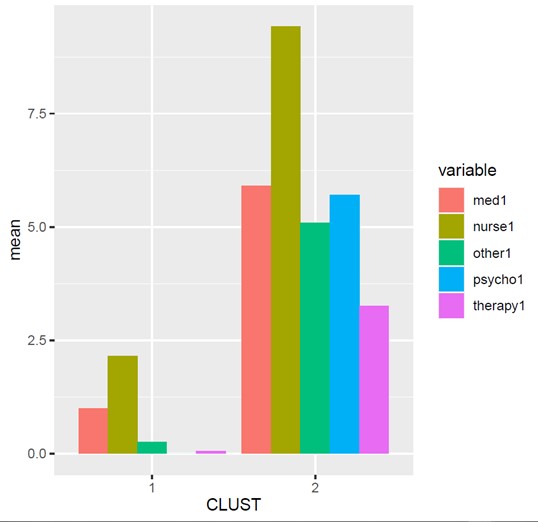


Figure S4: Service utilisation by latent profiles.


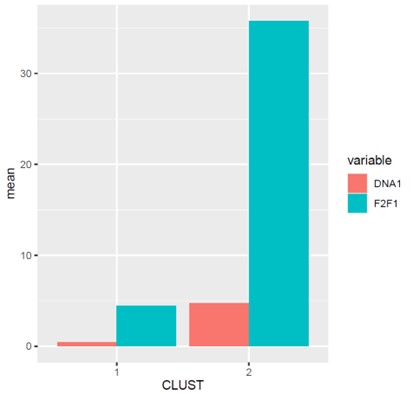

Supplement: Multimedia Appendix 3 [file ijmr_v14i1e55348_app3.docx]
