# Supplementary material for: Subtyping Service Receipt in Personality Disorder Services in South London: Observational Validation Study Using Latent Profile Analysis
Source: Interact J Med Res. 2025 Apr 15;14:e55348. doi: 10.2196/55348 (PMC12041827; doi:10.2196/55348)
Supplement: Multimedia Appendix 4 [file ijmr_v14i1e55348_app4.docx]

### Multimedia Appendix 4 – Data Cleaning procedures for categorical data

| Table S1: Ethnicity categories data cleaning procedure | | |  |
| --- | --- | --- | --- |
| ‘Raw’ unprocessed ethnicity categories | ‘Cleaned’, reduced categories | Final analytical categories |  |
| *White: British, Irish, Gypsy / Irish traveller, White other;* | White: British / non; | White |  |
| *Mixed race - West and Black Caribbean, Mixed race - West and Black African; Mixed race, White and Asian;*  *Mixed race – Other* | Mixed race | Mixed |  |
| *Asian British: Indian; Pakistani; Bangladeshi; Chinese; Other* | South Asian British; East Asian British; |  |  |
| *Black British - Caribbean; African; Other* | Black British | Black |  |
| *Non-specified categories: 'NA', Not known, Not specified; Not stated.*  *Other ethnic groups - Arab, other* | Other | Other |  |
| Table S2: Diagnostic categories data cleaning procedure | | | |
| ‘Raw’ unprocessed ICD-10 categories | ‘Cleaned’, reduced categories | Final analytical categories (DSM-IV) | |
| *F60 - Specific; Paranoid*  *F60.0 - Paranoid F60.1*  *F60.1 - Schizoid;*  *F60.2 - Dissocial*  *F60.3 - Emotionally unstable*  *F60.31 - Borderline Type*  *F60.4 - Histrionic. F60.4 - Histrionic*  *F60.5 - Anankastic; - F60.5 Anankastic*  *F60.6 - Anxious [avoidant]*  *F60.7 - Dependent; F60.7 - Dependent;*  *F60.8 - Other specific;*  *F60.9 Unspecified;*  *F61 -Mixed and other, Mixed & Other, Mixed; F61.x Mixed and other*  *F61.1 - Troublesome personality changes;* | *Paranoid*  *Schizoid*  *Dissocial*  *Emotional unstable Borderline*  *Histrionic*  *Anankastic*  *Anxious*  *Dependent*  *Other specified*  *Non specified*  *Mixed*  *Troublesome personality change* | A  B  C  other | |
|  | | | |
